# Glucocorticoids Selectively Inhibit Hippocampal CA1 Pyramidal Neurons Activity Through HCN Channels

**DOI:** 10.3390/ijms252211971

**Published:** 2024-11-07

**Authors:** Chenyang Li, Tongchuang Lu, Chengfang Pan, Changlong Hu

**Affiliations:** 1 Department of Physiology and Neurobiology, School of Life Sciences, Fudan University, Shanghai 200438, China; 2 International Human Phenome Institute (Shanghai), Shanghai 200433, China

**Keywords:** glucocorticoids, non-genomic effects, hyperpolarization-activated cyclic nucleotide-gated channel, hippocampal trisynaptic circuit

## Abstract

Glucocorticoids are known to influence hippocampal function, but their rapid non-genomic effects on specific neurons in the hippocampal trisynaptic circuit remain underexplored. This study investigated the immediate effects of glucocorticoids on CA1 and CA3 pyramidal neurons, and dentate gyrus (DG) granule neurons in rats using the patch-clamp technique. We found that a 5 min extracellular application of corticosterone significantly reduced action potential firing frequency in CA1 pyramidal neurons, while no effects were observed in CA3 or DG neurons. The corticosterone-induced inhibition in CA1 was blocked by the glucocorticoid receptor antagonist CORT125281, but remained unaffected by the mineralocorticoid receptor antagonist spironolactone. Notably, membrane-impermeable bovine serum albumin-conjugated dexamethasone mimicked corticosterone’s effects on CA1 neurons, which exhibited prominent hyperpolarization-activated cyclic nucleotide-gated (HCN) channel currents. Pyramidal neurons in CA3 and granular neurons in the DG showed little HCN channel currents. Corticosterone enhanced HCN channel activity in CA1 neurons via glucocorticoid receptors, and the HCN channel inhibitor ZD7288 abolished corticosterone’s suppressive effects on action potentials. These findings suggest that glucocorticoids selectively inhibit CA1 pyramidal neuron activity through HCN channels, providing new insight into the mechanisms of glucocorticoid action in hippocampal circuits.

## 1. Introduction

Produced by the zona fasciculata cells of the adrenal cortex in a diurnal pattern, glucocorticoids are widely used in synthetic form to treat various diseases, despite concerns about side effects, particularly for neurological disorders [1,2]. Understanding the molecular mechanisms of glucocorticoid action can help minimize these side effects. Known as stress hormones, glucocorticoids affect neurons through both genomic and non-genomic signaling pathways. The hippocampus, a key effector of glucocorticoids in the brain, also plays a crucial role in their secretion [3]. The discovery of glucocorticoid receptors in the hippocampus has advanced the study of stress and brain plasticity [4]. In the mammalian brain, prolonged increases in glucocorticoids lead to significant structural remodeling in the hippocampus [5], the medial prefrontal cortex, the orbital frontal cortex [6], and the amygdala [7] through genomic signaling pathways. However, the rapid non-genomic effects and mechanisms of glucocorticoids in the brain remain poorly understood.

Pyramidal neurons in CA1 and CA3 and granular neurons in the dentate gyrus (DG) make up the DG-CA3-CA1 trisynaptic circuit within the hippocampus. The trisynaptic circuit plays a crucial role in the formation and consolidation of memories [8]. In this study, we investigated the rapid effects of glucocorticoids on neurons within this circuit. Our results show that corticosterone rapidly inhibited action potential firing frequency in CA1 pyramidal neurons, but did not affect action potential firing frequency in CA3 pyramidal neurons and DG granular neurons. Corticosterone selectively inhibited the activity of hippocampal CA1 pyramidal neurons through HCN channels.

## 2. Results

### 2.1. Corticosterone Selectively Inhibits the Activity of CA1 Pyramidal Neurons in the Hippocampus

**Figure 1:**
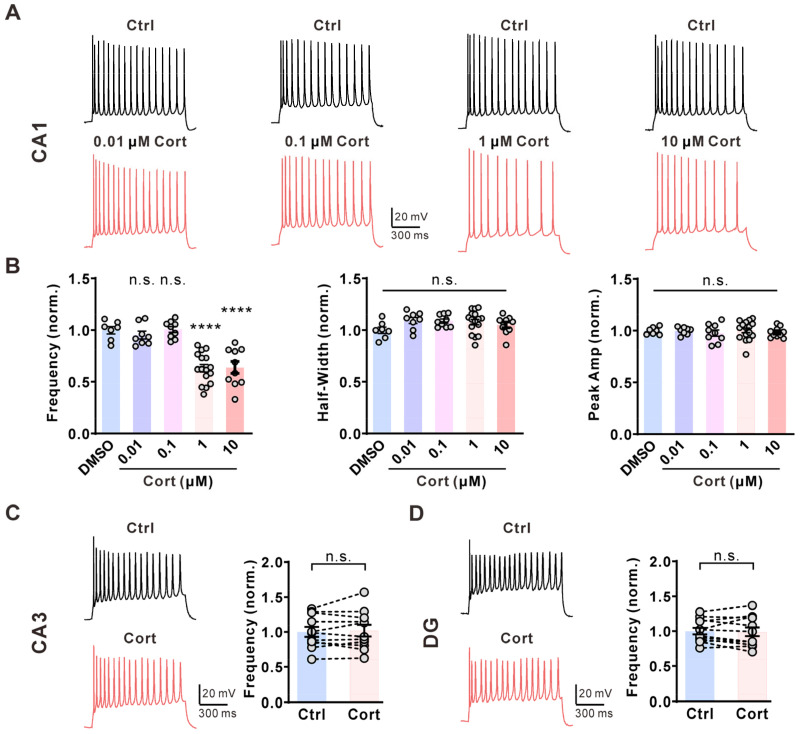
Corticosterone selectively inhibits the activity of CA1 pyramidal neurons in the hippocampus. (**A**) Representative action potential (AP) firings induced by 1 s, 60 pA current injection via a whole-cell configuration under the control condition (0.01% DMSO, **top**) and subsequently in the presence of 0.01 μM, 0.1 μM, 1 μM, or 10 μM corticosterone (Cort) (**bottom**) in the same CA1 pyramidal neuron for 5 min. (**B**) Statistics for the firing frequency, amplitude, and half-width from (**A**) (*n* = 7–16 for each concentration). The *p*-values were calculated using a one-way ANOVA with the Bonferroni post hoc test. **** *p* < 0.0001; n.s., not significant. (**C**) **Left**, representative AP firings induced by 1 s, 60 pA current injection via a whole-cell configuration under the control condition (0.01% DMSO, black) and subsequently in the presence of 1 μM Cort (red) in the same CA3 pyramidal neuron for 5 min. **Right**, statistics for AP firing frequency from **Left** using a two-tailed paired *t*-test (*n* = 11). n.s., not significant. (**D**) **Left**, representative AP firings induced by 1 s, 60 pA current injection via a whole-cell configuration under the control condition (0.01% DMSO, black) and subsequently in the presence of 1 μM Cort (red) in the same dentate gyrus (DG) granular neuron for 5 min. **Right**, statistics for AP firing frequency from **Left** using a two-tailed paired *t*-test (*n* = 12). n.s., not significant.

Brain slices from postnatal week 2 Sprague Dawley rats of either sex were used to examine the effects of glucocorticoids on the three major cell groups in the hippocampus. We first tested the effect of corticosterone on the activity of hippocampal CA1 pyramidal neurons in brain slices. In CA1 pyramidal neurons, repetitive action potential (AP) firings were elicited by the injection of a 1 s, 60 pA current via a whole-cell configuration. Extracellular application of corticosterone quickly inhibited AP frequency in CA1 pyramidal neurons in a dose-dependent manner without affecting AP half-width and peak amplitude (Figure 1A,B). The decrease in AP firings reached a plateau in the presence of 1 μM corticosterone in the extracellular solution (Figure 1B); therefore, we used 1 μM corticosterone for subsequent experiments. Next, we investigated whether corticosterone has a similar effect on neurons in CA3 and the DG region. Administration of corticosterone (1 μM) to the extracellular solution did not affect AP firing frequency in CA3 pyramidal neurons (Figure 1C) or DG granular neurons (Figure 1D).

### 2.2. Corticosterone Inhibits AP Firing Frequency via the Membrane-Associated Glucocorticoid Receptor

**Figure 2:**
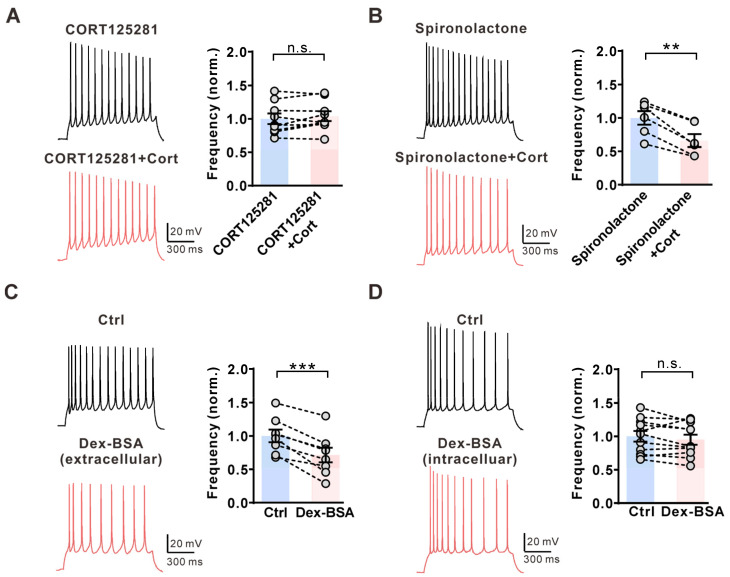
Corticosterone inhibits AP firing frequency via the membrane-associated glucocorticoid receptor. (**A**) **Left**, representative AP firings induced by 1 s, 60 pA current injection in the presence of the glucocorticoid receptor antagonist CORT125281 (10 μM, black) in the extracellular solution and subsequently in the presence of an additional 1 μM Cort (red) in the same CA1 pyramidal neuron. **Right**, statistics for AP firing frequency from **Left** using a two-tailed paired *t*-test (*n* = 9). n.s., not significant. (**B**) **Left**, representative AP firings induced by 1 s, 60 pA current injection in the presence of the mineralocorticoid receptor antagonist spironolactone (10 μM, black) in the extracellular solution and subsequently in the presence of an additional 1 μM Cort (red) in the same CA1 pyramidal neuron. **Right**, statistics for AP firing frequency from **Left** using a two-tailed paired *t*-test (*n* = 6). ** *p* < 0.01. (**C**) **Left**, Representative AP firings induced by 1 s, 60 pA current injection under the control condition (0.01% DMSO, black) and subsequently in the presence of 1 μM Dex-BSA (red) in the extracellular solution in the same CA1 pyramidal neuron. **Right**, statistics for AP firing frequency from **Left** using a two-tailed paired *t*-test (*n* = 8). *** *p* < 0.001. (**D**) Similar to C, but with 1 μM Dex-BSA in the pipette solution (*n* = 11). n.s., not significant.

Glucocorticoids normally act via glucocorticoid receptors or mineralocorticoid receptors. Preincubation with the glucocorticoid receptor inhibitor CORT125281 (10 μM, 20 min) blocked corticosterone-induced inhibition of AP firing in CA1 pyramidal neurons (Figure 2A), whereas the mineralocorticoid receptor inhibitor spironolactone (10 μM, 20 min) did not (Figure 2B). The rapid effect of glucocorticoids is normally mediated by membrane-associated receptors [9]. Therefore, we tested the effect of dexamethasone-BSA conjugate (Dex-BSA), which cannot pass through cell membranes, on AP firing. We found that extracellular application of 1 μM Dex-BSA had similar effects on AP firing as 1 μM corticosterone in CA1 pyramidal neurons (Figure 2C), but intracellular application of 1 μM Dex-BSA (in pipette solution) had no effects on AP firing (Figure 2D). These results suggested that corticosterone inhibits the activity of CA1 pyramidal neurons in the hippocampus through membrane-associated glucocorticoid receptors.

### 2.3. Corticosterone Increases I_h_ Amplitude Specifically in CA1 Pyramidal Neurons via Glucocorticoid Receptor

**Figure 3:**
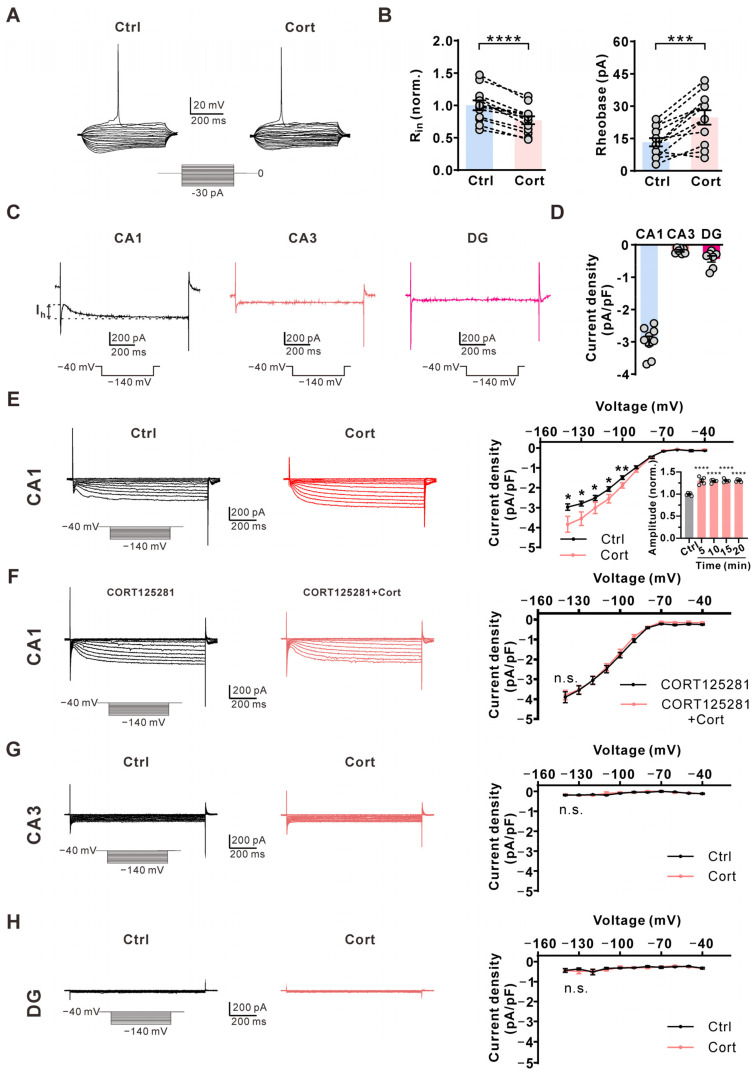
Corticosterone increases I_h_ amplitude specifically in CA1 pyramidal neurons via glucocorticoid receptor. (**A**) Representative membrane potential responses to the consecutive 3 pA step current injections starting from −30 pA under the control condition (0.01% DMSO, **left**) and subsequently in the presence of 1 μM corticosterone (Cort) (**right**) in the same CA1 pyramidal neuron. (**B**) Statistics for the input resistance (R_in_) and the amplitude of the injected current inducing the first action potential (Rheobase) from (**A**) using a two-tailed paired *t*-test (*n* = 12). *** *p* < 0.001; **** *p* < 0.0001. (**C**) Representative I_h_ traces induced by a hyperpolarization pulse from −40 to −140 mV in CA1 pyramidal neuron, CA3 pyramidal neuron, or DG granule neuron. (**D**) Statistics for the I_h_ amplitude from C (*n* = 7–9). (**E**) **Left**, representative I_h_ traces induced by a hyperpolarization pulse from −40 to −140 mV under the control condition (black) and subsequently in the presence of 1 μM Cort (red) in the same CA1 pyramidal neuron. **Right**, plot of the current–voltage relationship from **Left**. *p*-values were calculated using a two-tailed *t*-test (*n* = 8). * *p* < 0.05; ** *p* < 0.01. Effects of corticosterone on I_h_ at 5, 10, 15, and 20 min (insert). *p*-values were calculated using a one-way ANOVA with the Bonferroni post hoc test (*n* = 5). **** *p* < 0.0001. (**F**) **Left**, representative I_h_ traces induced by a hyperpolarization pulse from −40 to −140 mV in the presence of 10 μM CORT125281 in the extracellular solution (black) and subsequently in the presence of an additional 1 μM Cort (red) in the same CA1 pyramidal neuron. **Right**, statistics for the I_h_ amplitude from **Left** using a two-tailed paired *t*-test (*n* = 6). n.s., not significant. (**G**) **Left**, representative I_h_ traces induced by a hyperpolarization pulse from −40 to −140 mV under the control condition (black) and subsequently in the presence of 1 μM Cort (red) in the same CA3 pyramidal neuron. **Right**, plot of the current–voltage relationship from **Left** (*n* = 7). n.s., not significant. (**H**) **Left**, representative I_h_ traces induced by a hyperpolarization pulse from −40 to −140 mV under the control condition (black) and subsequently in the presence of 1 μM Cort (red) in the same DG granule neuron. **Right**, plot of the current–voltage relationship from **Left** (*n* = 7). n.s., not significant.

We then explored why corticosterone inhibited AP firing in CA1 but not CA3 pyramidal neurons or DG granular neurons. Hyperpolarization-activated and cyclic nucleotide-gated (HCN) channels play a key role in input resistance and neural activity control [10]. We found that 1 μM corticosterone significantly reduced the input resistance of CA1 pyramidal neurons, and increased the rheobase of AP firing in CA1 pyramidal neurons (Figure 3A,B), suggesting HCN channel involvement. Therefore, we investigated the HCN current (I_h_) in the neurons that make up the trisynaptic circuit within the hippocampus. The CA1 pyramidal neuron had obvious I_h_ currents, while CA3 pyramidal neurons and DG granular neurons had little I_h_ currents (Figure 3C,D). A 5 min extracellular application of corticosterone significantly increased I_h_ currents in CA1 pyramidal neurons (Figure 3E). Further incubation with corticosterone for 10, 15, and 20 min produced effects similar to those observed at 5 min (Figure 3E). This corticosterone-induced effect was abolished by the glucocorticoid receptor-specific inhibitor CORT125281 (Figure 3F). Corticosterone had no effects on the inward currents in CA3 pyramidal neurons (Figure 3G) and DG granular neurons (Figure 3H). These above data suggest that glucocorticoids inhibit AP firing frequency in CA1 pyramidal neurons via the HCN channel.

### 2.4. Corticosterone Inhibits AP Firing Frequency in CA1 Pyramidal Neurons via the HCN Channel

**Figure 4:**
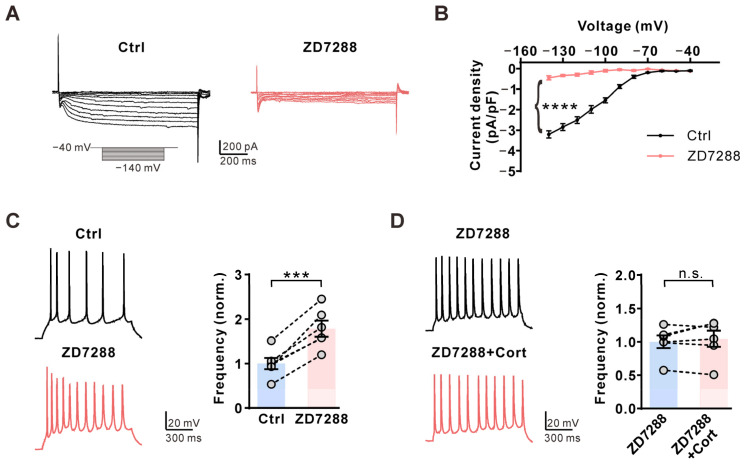
Corticosterone inhibits AP firing frequency via the HCN channel. (**A**) **Left**, representative I_h_ traces induced by a hyperpolarization pulse from −40 to −140 mV under the control condition (black) and subsequently in the presence of 10 μM ZD7288 (red) in the same CA1 pyramidal neuron. (**B**) Plot of the current–voltage relationship from A. *p*-values were calculated using a two-tailed *t*-test (*n* = 7). **** *p* < 0.0001. (**C**) **Left**, representative AP firings induced by 1 s, 30 pA current injection via a whole-cell configuration under the control condition (black) and subsequently in the presence of 10 μM ZD7288 (red) in the same CA1 pyramidal neuron. **Right**, statistics for the firing frequency from **Left** using a two-tailed paired *t*-test (*n* = 6). *** *p* < 0.001. (**D**) **Left**, representative AP firings induced by 1 s, 30 pA current injection via a whole-cell configuration in the presence of 10 μΜ ZD7288 in the extracellular solution (black) and subsequently in the presence of an additional 1 μM Cort (red) in the same CA1 pyramidal neuron. **Right**, statistics for the firing frequency from **Left** using a two-tailed paired *t*-test (*n* = 6). n.s., not significant.

Finally, we used ZD7288, the HCN specific inhibitor, to test whether the corticosterone-induced effect is mediated by HCN channels. Extracellular application of 10 μM ZD7288 nearly eliminated I_h_ currents in CA1 pyramidal neurons (Figure 4A,B) and significantly increased the AP frequency (Figure 4C). Preincubation with ZD7288 prevented the corticosterone-induced inhibition of AP firing in CA1 pyramidal neurons (Figure 4D). These results suggested that corticosterone selectively inhibits CA1 pyramidal neuron activity by increasing I_h_ currents.

## 3. Discussion

In this study, we demonstrated that corticosterone rapidly inhibited AP firing frequency in hippocampal CA1 pyramidal neurons, but did not affect AP firing frequency in hippocampal CA3 pyramidal neurons and hippocampal DG granular neurons. Four HCN channel subunits—HCN1, HCN2, HCN3, and HCN4—have been identified, and their expression in the hippocampus has been studied extensively. Previous studies have shown mRNA and protein expression of HCN1, HCN2, and HCN4 channels in both the mouse and rat hippocampus. Specifically, the expression of HCN channels in the CA1 region is much higher than the total expression in the CA3 and DG regions combined [11]. Although HCN protein is also expressed in CA3, patch-clamp recordings have shown that CA3 neurons display either small or undetectable I_h_ currents, in contrast to the higher I_h_ current density in CA1 neurons [12,13]. Our findings align with these previous studies, as we detected significant I_h_ currents only in CA1 pyramidal neurons. Our results indicate that corticosterone selectively inhibits CA1 pyramidal neuron activity by enhancing I_h_ currents. Previous studies showed that HCN channels inhibit EPSPs via interactions with the M-type K^+^ channel in mouse hippocampal CA1 neurons [14]. HCN channels in neurons mediate both excitatory and inhibitory effects based on the influence of I_h_ on the resting membrane potential (excitatory effect) and on input resistance (inhibitory effect) [10]. Increased I_h_ reduces input resistance and requires larger inputs to trigger APs. Accordingly, we found that corticosterone quickly increases I_h_ and reduces input resistance, and thus decreases the excitability of CA1 pyramidal neurons. A previous study reported that chronic stress reduces input resistance and decreases neuronal excitability in hippocampal CA1 neurons by increasing HCN channel expression [15]. Together, corticosterone can enhance I_h_ through both genomic and non-genomic pathways, thereby reducing the activity of hippocampal CA1 neurons.

Previous studies have indicated that the non-genomic effects of glucocorticoids are likely mediated by glucocorticoid receptors located on the cell membrane [9]. Our findings suggest that corticosteroids enhance HCN currents through these membrane-associated glucocorticoid receptors. Research has shown that glucocorticoids exert their rapid effects through several downstream signaling pathways, including PKA, PKC, and ERK [16,17,18]. Ffrench-Mullen reported that cortisol rapidly inhibits L-type calcium channels in hippocampal pyramidal neurons via the PKC signaling pathway rather than through PKA [19]. Our previous study demonstrated that cortisol increases Kv2.2 channel currents by activating ERK1/2 in cortical neurons [20]. Moreover, glucocorticoids have been shown to regulate Kv1.3 channels, possibly through direct interaction with the channels in parvocellular neurons [21].

HCN channels are primarily regulated by cAMP in a direct, PKA-independent manner [10]. In addition to cAMP, various factors can activate HCN channels, including phosphatidylinositol-4,5-bisphosphate [22], PKA [23], Src kinase [24,25], and other cyclic nucleotides [26]. These factors exert different effects on the HCN1, HCN, and HCN4 subunits. For example, cAMP shifts the activation curve of HCN currents to more positive potentials, with the shift in V_1/2_ varying by subtype and conditions: between 10 and 25 mV for HCN2 and HCN4, but only 2–6 mV for HCN1 [26]. Importantly, we did not observe a significant change in V_1/2_, suggesting that corticosteroids may not influence HCN channels through the cAMP pathway. Further investigation is warranted to elucidate the specific HCN channels involved in the inhibitory effects of corticosteroids on CA1 neuron activity, as well as the signaling pathways that mediate this process.

Neuronal excitability is sensitive to HCN channel modulation. Previous research demonstrated that even modest increases in HCN currents can have pronounced physiological effects. For instance, Garratt et al. reported that a 20% increase in HCN currents induced by serotonin significantly affected the excitability of rat facial motoneurons [27]. Gasparini and DiFrancesco found that a ~29% increase in HCN currents in rat CA1 neurons corresponded with a notable reduction in excitability [28]. Additionally, Herrmann et al. showed that a 30% increase in HCN currents in DRG neurons had significant implications for inflammatory pain behaviors [23]. Moreover, a comparable increase in HCN currents in CA1 neurons has been linked to chronic social defeat stress, impacting social avoidance and spatial working memory [15]. In the ventral tegmental area, a 33% increase in HCN currents was associated with morphine-induced conditioned place preference [29], and similar changes in dopamine neurons have been implicated in cocaine reinforcement [30].

Hippocampal trisynaptic circuitry is essential for spatial and episodic memory formation. A sustained increase in glucocorticoids results in dendritic retraction in the CA3 region and reduced neurogenesis in the dentate gyrus [31], which are associated with memory impairments [32]. Glucocorticoid levels exhibit distinct circadian and ultradian rhythms within the brain. Recent research [33] indicates that the ultradian rhythmicity of glucocorticoids plays a crucial role in regulating normal emotional and cognitive responses in humans. During these fluctuations, glucocorticoids can rapidly modulate neural function through non-genomic effects. Here, we showed that corticosterone quickly inhibits the excitability of hippocampal CA1 neurons, which may also contribute to glucocorticoid-induced memory loss. Moreover, rapid corticosteroid action in the hippocampus may also contribute to the rapid negative feedback regulation of the hypothalamic–pituitary–adrenal axis [34].

In conclusion, our results demonstrated that glucocorticoids selectively inhibit the activity of hippocampal CA1 pyramidal neurons through HCN channels, and provide new insights into the rapid effects of glucocorticoids on the brain.

## 4. Materials and Methods

### 4.1. Animal and Slice Preparation

Brain slices containing hippocampus samples were prepared from Sprague Dawley rats of either sex from postnatal day 14. Rats were anesthetized using pentobarbital sodium and decapitated. The whole brain was transferred into ice-cold cutting solution containing (in mM): 220 sucrose, 3 KCl, 5 MgCl_2_, 1 CaCl_2_, 1.25 NaH_2_PO_4_, 26 NaHCO_3_, and 10 glucose. For electrophysiological recordings, 250 μm coronal slices were prepared using a vibratome (VT1200s, Leica, Wetzlar, Germany). Slices were incubated for 40 min at 35 °C in incubation solution containing (in mM): 125 NaCl, 26.2 NaHCO_3_, 2.5 KCl, 1.5 MgSO_4_, 1 NaH_2_PO_4_, 2.5 CaCl_2_, and 11 glucose (95% O_2_/5% CO_2_).

### 4.2. Electrophysiology

Action potential in hippocampal neurons was recorded using a Multiclamp 700B amplifier (Molecular Devices, San Jose, CA, USA). The extracellular solution contained (in mM): 125 NaCl, 26.2 NaHCO_3_, 2.5 KCl, 1.5 MgSO_4_, 1 NaH_2_PO_4_, 2.5 CaCl_2_, and 11 glucose (95% O_2_/5% CO_2_). The pipette (4–6 MΩ) solution contained (in mM): 150 K-gluconate, 8 NaCl, 10 HEPES, 2 Mg-ATP, 0.1 Na_3_-GTP, and 0.4 EGTA, with a pH of 7.3 adjusted with KOH. Action potential was sampled at 10 kHz and filtered at 2 kHz, and a 1 s, 60 pA current was injected to induce AP trains.

Measurements of I_h_ in hippocampal neurons were taken with a sampling rate of 5 kHz and a filtering rate of 2 kHz using a Multiclamp 700B amplifier (Molecular Devices, San Jose, CA, USA). The extracellular solution contained (in mM): 125 NaCl, 3 KCl, 1.25 NaH_2_PO_4_, 25 NaHCO_3_, 2 CaCl_2_, 1 MgCl_2_, 12.5 glucose, 1 BaCl_2_, and 10 TEA-Cl (95% O_2_/5% CO_2_). The pipette (4–6 MΩ) solution contained (in mM): 120 K-gluconate, 20 KCl, 10 HEPES, 4 NaCl, 7 Na_2_-phosphocreatine, 4 Mg-ATP, and 0.3 Na_3_-GTP, with a pH of 7.3 adjusted with KOH.

Corticosterone (HY-B1618 MCE, Trenton, NJ, USA), CORT125281 (HY-117880 MCE, Trenton, NJ, USA), and ZD7288 (HY-101346 MCE, Trenton, NJ, USA) were purchased from MCE. BSA-Dexamethasone was purchased from Xi’an ruixi biological Technology Company (Xi’an, China). Spironolactone (S4054, Selleck, Houston, TX, USA) was purchased from Selleck Chemicals.

### 4.3. Statistical Analysis

Data analysis was performed with Clampfit 10.7 (Axon Instruments, Foster City, CA, USA) and GraphPad Prism (v9.4, GraphPad Software Inc, San Diego, CA, USA). Two-tailed paired *t*-tests were used to compare two samples, and one-way ANOVA with the Bonferroni post hoc test was used to compare multiple samples. Data are given as means ± S.E.M, and n indicates the number of tested cells. *p* < 0.05 was considered statistically significant.

## Data Availability

All data used during this study are available from the corresponding author upon request.
